# Resilience and self-compassion affect selfhandicapping in Turkish undergraduate nursing students: A correlational study

**DOI:** 10.7705/biomedica.7578

**Published:** 2025-03-28

**Authors:** Sinem Yalnızoğlu Çaka, Sümeyra Topal

**Affiliations:** 1 Department of Pediatric Nursing, Kocaeli University, Kocaeli, Turkey Kocaeli University Department of Pediatric Nursing Kocaeli University Kocaeli Turkey; 2 Department of Pediatric Nursing, Kahramanmaraş istiklal University, Kahramanmaraş, Turkey Kahramanmaras Sütçü Imam University Department of Pediatric Nursing Kahramanmaraş istiklal University Kahramanmaraş Turkey

**Keywords:** Students, nursing, resilience, psychological, self-compassion., estudiantes de enfermería, resiliencia psicológica, autocompasión

## Abstract

**Introduction.:**

Psychological resilience and self-compassion are qualities that nurses should have when helping people with health problems.

**Objective.:**

To determine the effect of resilience on self-handicapping and self-compassion in nursing students.

**Materials and methods.:**

This research has a correlational design. The study sample included nursing students who met the inclusion criteria (n = 369). Data were collected using the Connor-Davidson Resilience Scale, Self-Handicapping Scale, and Self-Compassion Scale questionnaires.

**Results.:**

The questionnaire scores of the nursing students were above the average, with 63.91 ± 14.54 for the Connor-Davidson Resilience Scale and 82.68 ± 11.32 for the SelfHandicapping Scale; their self-compassion level was high, with a mean of 13.92 ± 2.87 points on the Self-Compassion Scale. We found a significant negative correlation between the mean scores of the Connor-Davidson Resilience Scale (r = -0.409; p = 0.000) and the Self-Compassion Scale (r = -0.524; p = 0.000) with the Self-Handicapping Scale. We also obtained a positive and significant correlation between the mean scores of the Connor-Davidson Resilience Scale and the Self-Compassion Scale (r = 0.486; p = 0.000). According to the regression analysis, the effect of these two scales on the Self-Compassion Scale was 30.2%.

**Conclusions.:**

Considering the study results, we can argue that as the students’ resilience and self-compassion increase, their tendency to self-handicap decreases. For health professionals and patients’ safety, it is very important to determine the levels of resilience, self-handicapping, and self-compassion because these factors may increase anxiety and stress in nursing students, affecting the proper care of patients during the work period.

## Resumen

**Introducción.:**

La resiliencia psicológica y la autocompasión son cualidades fundamentales de los enfermeros para que puedan ayudar eficazmente a las personas con problemas de salud.

**Objetivo.:**

Determinar el efecto de la resiliencia en el autosabotaje y la autocompasión en estudiantes de enfermería.

**Materiales y métodos.:**

Esta investigación tiene un diseño correlacional. La muestra del estudio incluyó estudiantes de enfermería que cumplían los criterios de inclusión (n = 369). Los datos se recopilaron mediante los cuestionarios escala de resiliencia de ConnorDavidson, escala de autosabotaje y escala de autocompasión.

**Resultados.:**

Los resultados de los cuestionarios aplicados a los estudiantes de enfermería estuvieron por encima del promedio con 63,91 ± 14,54 puntos en la escala de resiliencia de Connor-Davidson y 82,68 ± 11,32 en la escala de autosabotaje. Su nivel de autocompasión fue alto, con un promedio de 13,92 ± 2,87 puntos en la escala de autocompasión. Hubo una correlación negativa y significativa entre las puntuaciones medias obtenidas en la escala de resiliencia de Connor-Davidson (r = -0,409; p = 0,000) y en la escala de autocompasión (r = -0,524; p = 0,000) con el puntaje de la escala de autosabotaje. Hubo una correlación positiva y significativa entre las puntuaciones medias obtenidas por los participantes del estudio en la escala de resiliencia de Connor-Davidson y la escala de autocompasión (r = 0,486; p = 0,000). Según el análisis de regresión, el efecto de los puntajes obtenidos en estas dos escalas sobre el autosabotaje fue del 30,2 %.

**Conclusiones.:**

Los resultados de este estudio sugieren que a medida que aumenta la resiliencia de los estudiantes, aumenta su autocompasión y disminuye su tendencia al autosabotaje. Para los profesionales de la salud y por la seguridad de los pacientes es importante determinar los niveles de resiliencia, autocompasión y autosabotaje porque estos pueden incrementar la ansiedad y el estrés de los estudiantes de enfermería y, por lo tanto, afectar el cuidado adecuado de los pacientes durante la jornada laboral.

Resilience is defined as the ability to successfully overcome negative situations and adapt to stressful circumstances despite difficult conditions ^1^. It is also the ability of an individual to turn difficulties into opportunities and to learn while struggling with challenging situations ^2^.

Resilience helps students to recover quickly, adapt, and maintain their well-being when facing stressful and challenging life conditions ^1^. If students do not have enough strength and resources to cope with the daily stress or difficulties, they may experience failure and may feel powerless to deal with obstacles ^3^^,^^4^. People resort to different strategies to manage the struggles that negatively affect their lives, like self-handicapping. Self-handicapping individuals try to protect themselves by internalizing their successes and externalizing their failures ^5^. Such a strategy, which seems functional in the short term, may lead to negative psychological consequences in the long term.

Increasing awareness of an individual’ strengths and competencies, strengthening psychological resilience, and improving problem-solving skills will help prevent self-handicapping behaviors ^5^^,^^6^. At this stage, it is important for nurses to accept themselves and be self-compassionate ^6^. Self-Compassion involves approaching events without judging oneself when erring or experiencing failures and being emotionally supportive and understanding toward oneself ^7^. Due to their profession, nurses are intensely affected by the pain and distress of people. Compassion has been reported as a fundamental component of professional nursing care ^8^. However, compassionate care can only be provided by showing compassion to oneself ^9^.

Improving and increasing resilience and self-compassion levels of nursing students -who will work as health professionals- will positively affect their health and that of the people they care for ^8^^,^^9^. Thus, it is very important to investigate self-handicapping and self-compassion states of nursing students and to define the relationship between these concepts and resilience. Accordingly, our purpose was to determine the effect of resilience on selfhandicapping and self-compassion of nursing students.

## Materials and methods

### Study design

This research has a correlational design. The STROBE (Strengthening the Reporting of Observational Studies in Epidemiology) guideline was used to report our results.

### Time and place of study

The study was conducted between December 2021 and January 2022 with nursing students of istiklal University in the Kahramanmaraş province in the southwest region of Turkey.

### Study population

The study population comprised all students enrolled in the nursing department of the istiklal University (N = 425). Selected individuals had to be 18 years or older, voluntarily agree to participate in the study at the time of data collection and not have any chronic or psychiatric disorders previously diagnosed. Nine individuals who did not finish the questionnaires were excluded. The data of 369 students were included in the study.

### Data collection tools

Data were collected using a questionnaire developed by the authors, along with three others: the Connor-Davidson Resilience Scale, Self-Handicapping Scale, and Self-Compassion Scale. The authors provided participants with data collection forms to complete. The data collection process took approximately 20 minutes.

*Authors’ questionnaire:* The authors developed 16 questions in line with literature reports covering the socio-demographic characteristics of students. These questions addressed their age, year of study, educational status, employment status, economic level, etc.

*Connor-Davidson Resilience Scale (CD-RISC):* The scale developed by Connor and Davidson to measure resilience includes 25 items in the original form ^1^. Karairmak performed the Turkish validity and reliability study of the scale ^10^. The scale is a five-point Likert-type scale. Each question on the scale is scored between zero and four points. The minimum score that can be obtained is 0 and the maximum score is 100. The Cronbach’s alpha reliability coefficient of the scale for this study was 0.90.

*Self-Handicapping Scale:* The scale was developed by Jones and Rhodewalt ^11^, and its Turkish validity and reliability were conducted by Akin ^7^. The scale consists of 25 items and is a six-point Likert-type scale. The scores obtained vary between 25 to 150. Cronbach’s alpha reliability coefficient of the scale for this study was 0.70.

*Self-Compassion Scale:* The scale was developed by Neff ^12^, and the Turkish validation and reliability of the 26-item scale were carried out by Akin *et al*^7^. The scale is a five-point Likert-type scale. It has six sub-dimensions: self-kindness, self-judgment, common humanity, isolation, mindfulness, and over-identification. A score of 1 to 2.5 points indicate low self-compassion, 2.5 to 3.5 points indicate medium self-compassion, and 3.5 to 5 points indicate high self-compassion. Cronbach’s alpha reliability coefficient of the scale for this study was 0.90.

### Data analysis

In the study, data of 369 participants were assessed and analyzed with the IBM software SPSS Statistics™, version 23. The descriptive characteristics of the participants were analyzed using frequency (%) for categorical variables and mean and standard deviation (SD) for continuous variables. The independent sample t test and the one-way analysis of variance (ANOVA) were applied for categorical variables. For continuous variables, the associations were evaluated using Pearson’s correlation. The correlation values between 0.00 and 0.29 suggested a weak correlation, 0.30 to 0.70 indicated a moderate correlation, 0.71 to 0.99 demonstrated a strong correlation, and 1.00 showed a very strong correlation ^13^. The results were interpreted using a multiple linear regression model to examine effects. Afterwards, a series of regression models were applied to test the mediating effect of psychological resilience and self-compassion on self-handicapping. A p value less than 0.05 was taken as the significance level.

### Ethical consideration

Before the study was conducted, ethics committee approval was received from the Faculty of Medicine Ethics Committee of Kahramanmaraş University on October 15, 2021 (approval number: 319). The necessary institutional permission was granted from the relevant university administration (No.: E-37919919-100- 88976). After informing the participants about the purpose of the study, where and how the data would be used, and the confidentiality of the answers, the students who signed the informed consent were included in the study.

## Results

The mean age of the students included was 20.50 ± 1.49. The data on descriptive characteristics of the 369 participants of the study are presented in table 1.

**Table 1:** Participant demographic characteristics (N = 369)

Variables		n	%	CD-RISC	SHS	Self-compassion Scale
				Mean ± SD	Mean ± SD	Mean ± SD
Gender						
	Female	277	75.1	63.77 ± 14.40	82.69 ± 11.33	13.94 ± 3.08
	Male t/p	92	24.9	64.30 ± 15.05 -0.299/0.765	82.63 ± 11.34 0.049/0.961	13.86 ± 2.11 0.220/0.826
Class						
	1^st^	88	23.8	66.78 ± 15.63	78.61 ± 11.83	14.62 ± 2.75
	2^nd^	87	23.6	62.83 ± 14.40	83.29 ± 11.87	13.95 ± 2.88
	3^rd^	109	29.5	62.71 ± 13.94	84.53 ± 10.37	13.76 ± 2.68
	4^th^	85	23.0	63.56 ± 14.12	83.88 ± 10.49	13.37 ± 3.11
	F/p			1.570/0.196	5.345/0.001^**abc^	2.919/0.034^*c^
Perceived economic level						
	Income is more than expenses	45	12.2	69.46 ± 17.02	78.82 ± 12.84	14.69 ± 2.58
	Income is equivalent to expenses	291	78.9	63.78 ± 13.67	82.88 ± 11.00	13.90 ± 2.96
	Income is less than expenses	33	8.9	57.48 ± 15.96	86.12 ± 10.73	13.07 ± 2.12
	F/p			6.715/0.001^**ab^	4.259 /0.015^*b^	3.125 /0.045^*b^
Place of residence						
	Village	63	17.1	60.61 ± 12.85	82.38 ± 11.32	13.61 ± 2.45
	Town	67	18.2	65.28 ± 17.11	82.37 ± 10.74	14.40 ± 2.69
	City	239	64.8	64.39 ± 14.11	82.84 ± 11.51	13.86 ± 3.01
	F/p			2.054/0.130	0.072/0.931	1.337/0.264
Family type						
	Nuclear family	292	79.1	63.53 ± 14.72	82.51 ± 11.31	13.99 ± 2.80
	Extended family	58	15.7	65.77 ± 14.08	83.51 ± 10.71	13.81 ± 3.13
	Fragmented family	19	5.1	64.00 ± 13.45	82.68 ± 13.58	13.21 ± 3.10
	F/p			0.573/0.564	0.189/0.828	0.692/0.501
Employment status						
	Working	29	7.9	68.51 ± 13.07	80.37 ± 12.64	14.68 ± 2.79
	Not working	340	92.1	63.51 ± 14.61	82.87 ± 11.20	13.85 ± 2.87
	t/p			1.782/0.076	1.141/0.255	1.499/0.135
Mother’s education level						
	Primary/Lower secondary	287	77.8	64.02 ± 14.14	82.38 ± 10.90	14.01 ± 2.76
	High school	54	14.6	66.64 ± 14.93	83.03 ± 12.59	13.97 ± 3.09
	University	28	7.6	57.46 ± 16.38	85.00 ± 13.01	12.86 ± 3.37
	F/p			3.769/0.020^*a,b^	0.710/0.492	2.078/0.127
Father’s education level						
	Primary/Lower secondary	204	55.3	63.12 ± 13.24	83.37 ± 10.26	13.81 ± 2.68
	High school	90	24.4	64.90 ± 14.92	80.44 ± 13.05	14.02 ± 3.47
	University	75	20.3	64.88 ± 17.30	83.48 ± 11.62	14.09 ± 2.58
	F/p			0.676/0.509	2.340/0.098	0.347/0.707
Willingly choose the profession						
	Yes	133	36.0	67.19 ± 12.72	80.05 ± 10.54	14.52 ± 2.65
	No	236	64.0	62.05 ± 15.19	84.16 ± 11.49	13.58 ± 2.94
	t/p			3.300/0.001^**^	-3.394/0.001^**^	3.051/0.002^**^
Status of receiving social support						
	Yes	262	71.0	66.34 ± 13.53	81.29 ± 11.57	14.47 ± 2.76
	No	107	29.0	57.95 ± 15.27	86.07 ± 9.94	12.58 ± 2.69
	t/p			5.202/0.000^**^	-3.745/0.000^**^	5.965/0.000^**^

CD-RISC: Connor-Davidson Resilience Scale; SHS: Self-Handicapping Scale; F: One-way ANOVA test; t: Independent sample t test; p: p value

a: 1-2, b: 1-3, c: 1-4

*p < 0.05

**p ≤ 0.001

No significant differences were found between gender, place of residence, family type, employment status, and father’s education level with the scales’ scores. Also, no significant differences were found between the year of study and the CD-RISC scale. We found statistically significant differences between the Self-Handicapping and Self-Compassion Scale (p < 0.05). Accordingly, first-year students had significantly lower CD-RISC scores than those of the second, third, and fourth year. In addition, the mean scores of the first year students on the Self-Compassion Scale were significantly higher than those of the fourth year (table 1).

We found a statistically significant difference between the perceived economic level and all three scales (p < 0.05). Accordingly, the CDRISC score averages of those with more income than their expenses are significantly higher than those with equal and lesser income. In addition, those with more income than their expenses have a lower mean score on the Self-Handicapping Scale and a significantly higher mean score on the Self-Compassion Scale. No significant relationship was found between the maternal education status of the students and their mean scores on the SelfHandicapping and Self-Compassion scales. However, a statistically significant difference was found between maternal education status and the CD-RISC score (p < 0.05). Thus, while the mean scores of the students with mothers having a primary or secondary education level were significantly lower than those with mothers having a high school education level, their scale mean scores were significantly higher than those with mothers with a university education level.

When examining the relationship between the scales with the voluntary choice of profession and perceiving social support of the students participating in the research, we identified a statistically significant difference with all three scales (p < 0.05). Accordingly, the mean scores of the CDRISC and Self-Compassion Scale for students who voluntarily chose their profession and perceived social support were significantly higher than those of the other group. However, their mean scores on the Self-Handicapping Scale were significantly lower (table 1).

The results of the study revealed that the students had a high level of resilience with a mean of 63.91 ± 14.54 points on the CD-RISC scale, a high tendency to self-handicapping with a mean of 82.68 ± 11.32 points on the Self-handicapping Scale, and a high level of self-compassion with a mean of 13.92 ± 2.87 points on the Self-Compassion Scale. We identified a negative and significant relationship between CD-RISC scores and tenacity and personal competence, tolerance of negative effect, and tendency toward spiritual items (p < 0.05). Subsequently, we could argue that as the tenacity of students increases, their tendency to self-handicap decreases.

We found a positive correlation between the mean score of the CD-RISC and that of the Self-Compassion Scale and its sub-dimensions (p < 0.05) (table 2). Thus, we interpret that as the tenacity of students increases, their self-compassion also increases. Similarly, we observed a significant negative correlation between the mean scores of the Self-Handicapping and the Self-Compassion scales and its sub-dimensions of self-kindness, self-judgment, common humanity, isolation, mindfulness, and over-identification (p < 0.05). Considering this result, we could affirm that as the self-care of students increases, their tendency to self-handicap decreases (table 2).

**Table 2:** Relationships between scales and sub-dimensions

		CD-RISC	Tenacity and personal competence	Tolerance of negative affect	Tendency toward spirituality	SHS	Self-compassion Scale	Self-kindness	Self-judgment	Common humanity	Isolation	Mindfulness	Over-identification
CD-RISC	r	1	0.954	0.848	0.566	-0.409	0.486	0.409	0.320	0.260	0.329	0.451	0.373
	p		0.000**	0.000**	0.000**	0.000**	0.000**	0.000**	0.000**	0.000**	0.000**	0.000**	0.000**
Tenacity and	r		1	0.700	0.398	-0.455	0.476	0.399	0.342	0.229	0.312	0.446	0.362
personal	p			0.000**	0.000**	0.000**	0.000**	0.000**	0.000**	0.000**	0.000**	0.000**	0.000**
competence													
Tolerance of	r			1	0.384	-0.315	0.439	0.364	0.259	0.217	0.333	0.396	0.365
negative affect	p				0.000**	0.000**	0.000**	0.000**	0.000**	0.000**	0.000**	0.000**	0.000**
Tendency toward	r				1	-0.053	0.182	0.171	0.066	0.222	0.101	0.172	0.100
spirituality	p					0.309	0.000**	0.001	0.206	0.000**	0.054	0.001	0.054
SHS	r					1	-0.524	-0.285	-0.519	-0.017	-0.533	-0.323	-0.544
	p						0.000**	0.000**	0.000**	0.746	0.000**	0.000**	0.000**
Self-compassion	r						1	0.755	0.786	0.530	0.754	0.747	0.793
Scale	p							0.000**	0.000**	0.000**	0.000**	0.000**	0.000**
Self-kindness	r							1	0.379	0.537	0.324	0.725	0.397
	p								0.000**	0.000**	0.000**	0.000**	0.000**
Self-judgment	r								1	0.132	0.716	0.347	0.707
	p									0.011*	0.000**	0.000**	0.000**
Common	r									1	0.130	0.527	0.163
humanity	p										0.012*	0.000**	0.002**
Isolation	r										1	0.352	0.691
	p											0.000**	0.000**
Mindfulness	r											1	0.434
	p												0.000**
Over-	r												1
identification	p												

CD-RISC: Connor-Davidson Resilience Scale; SHS: Self-Handicapping Scale; r: Pearson correlation analysis

*p < 0.05

**p < 0.001

As a result of the regression analysis evaluating the effects of the CDRISC scale and its sub-dimensions on self-handicapping, we found a statistically significant variance (p < 0.000), explained by two sub-dimensions (tenacity and personal competence with a tendency toward spirituality). When the CD-RISC sub-dimensions were considered together, their effect on the variance of the Self-Handicapping Scale scores was 22%. The regression analysis between the Self-Compassion Scale sub-dimensions on self-handicapping showed a statistically significant variance (p < 0.05), explained by five sub-dimensions (self-judgment, common humanity, isolation, mindfulness, and over-identification). When the Self-Compassion Scale sub-dimensions were considered together, their effect on the variance of the Self-Handicapping Scale scores was 36.9%. The variance explained by the CD-RISC and Self-Compassion Scale total scores regarding the Self-Handicapping Scale was statistically significant. According to the analysis, the effect of the CD-RISC and Self-Compassion Scale scores on the variance of the Self-Handicapping Scale scores was 30.2% (p = 0.000) (table 3).

**Table 3:** Multivariate regression analysis model for Self-Handicapping Scale

	Model	Coeficient	SE	β	t	^p^
CD-RISC sub-dimensions	(Constant)^a^	100.198	2.642 --		37.924	0.000**
	Tenacity and personal competence	-0.588	0.077	-0.499	-7.592	0.000**
	Tolerance of negative affect	-0.062	0.161	-0.025	-0.384	0.701
	Tendency toward spirituality	0.664	0.218	0.155	3.052	0.002**
Self-Compassion Scale sub-dimensions	(Constant)^b^	106.034	2.437	--	43.515	0.000**
	Self-kindness	-0.142	0.176	-0.052	-0.808	0.420
	Self-judgment	-0.360	0.157	-0.152	-2.298	0.022*
	Common humanity	0.586	0.172	0.174	3.415	0.001**
	Isolation	-0.648	0.189	-0.221	-3.435	0.001**
	Mindfulness	-0.501	0.217	-0.148	-2.310	0.021*
	Over-identification	-0.649	0.187	-0.227	-3.477	0.001**
Total Score	(Constant)^c^	116.101	2.704	--	42.940	0.000**
	CD-RISC	-0.158	0.039	-0.203	-4.059	0.000**
	Self-compassion Scale	-1.677	0.197	-0.425	-8.519	0.000**

CD-RISC: Connor-Davidson Resilience Scale; SE: Standard error

* p < 0.05

**p < 0.001

a R = 0.476, R2 = 0.227, Adjusted R2= 0.220, p = 0.000

b R = 0.616, R2 = 0.380, Adjusted R2= 0.369, p = 0.000

a R = 0.553, R2 = 0.305, Adjusted R2= 0.302, p = 0.000

## Discussion

Students need protective factors such as resilience and self-compassion to effectively cope with stressful situations, maintain logical awareness, and protect their physical and psychological health ^14^^,^^15^. Studies conducted with nursing students have shown that these individuals often struggle to use effective coping strategies to face difficult circumstances ^6^^,^^14^^-^^16^ and, in turn, may resort to self-handicapping behaviors ^5^. This study aimed to determine the relationship between resilience, self-handicapping strategies, and self-compassion levels in nursing students responsible for providing health care.

While no significant differences were found between the year of study and the CD-RISC scale, we identified a statistically significant difference between the Self-Handicapping and the Self-Compassion Scale. Some related studies reported that students’ mean scores on CD-RISC increase as they approach their senior year ^15^^,^^17^. However, other reports support the findings of this study ^3^^,^^18^, which found no significant relationship. Although no difference regarding resilience was determined between first- and fourth-year students, the decrease in mean CD-RISC scores revealed the need to review the current program curriculum and offer more psychological support to students.

When comparing the year of study with the mean scores on the SelfHandicapping Scale, we noted that the first-year students’ scores were significantly lower. In other studies, the authors stated that nursing education is more stressful for students than other health disciplines, particularly in the third and fourth years, when students’ stress levels are higher ^19^^,^^20^. It is possible that the intensification of vocational course content and clinical practices, as students advance in the program, causes an increase in stress levels ^19^^,^^21^^,^^22^. Unlike the results of this study, others reported no significant differences between the year of study and the Self-Handicapping Scale mean score ^16^^,^^20^.

The comparison between the year of study of nursing students and their Self-Compassion Scale scores showed that the mean scores of first-year students were significantly higher than those of senior students. This finding is congruent with the work of Nazik and Arslan, who also examined the self-compassion of nursing students ^23^. In the study conducted by Bulduk *et al*., with nursing students, since no fourth-year students were included in the evaluated population, the authors did not make a comparison for this year of study ^14^.

We observed that resilience and self-compassion were higher among nursing students with good economic status. Similar studies conducted with nursing students emphasized that perceived economic situation affects an individual’s psychological health ^21^^,^^24^. In addition, we found that the Self-Handicapping Scale mean scores of the students with income exceeding their expenses were lower than those of students with income below their expenses. In contrast, other studies reported no significant differences between the income level perceived by the students and their selfhandicapping mean scores ^16^^,^^25^.

According to this study’s findings, the CD-RISC and Self-Compassion Scale mean scores of the students who chose their profession voluntarily were significantly higher than those of the other group, and their SelfHandicapping Scale mean scores were significantly lower. Nursing encompasses the understanding of loving and helping people, requires resilience to stressful life events, and should be consciously selected ^3^^,^^21^. In similar studies on this subject, the students dissatisfied with their career choice had high levels of self-handicapping ^16^^,^^26^^,^^27^. Doing work with love and willingness increases the quality of the labor by enhancing self-esteem. People who love their professions may differ in their resilience and coping strategies when faced with difficulties ^5^^,^^28^.

We found that the mean scores of the CD-RISC and Self-Compassion Scale were significantly higher in nursing students with perceived social support. However, their Self-Handicapping Scale mean scores were significantly lower than those of the other group. Some studies have shown that social support is a buffering factor for stress and positively affects a person’s physical and psychological health by reducing the effects of stress ^29^^,^^30^. Likewise, we identified a strong and positive relationship between psychological resilience levels and perceived social support ^3^^,^^31^. However, studies like the one of Şahin *et al.* (2017) did not find a relationship between the psychological resilience of nursing students and social support ^15^.

Regarding the Self-Compassion Scale, the mean scores of the nursing students with perceived social support were significantly higher than those of the other group. In the published literature, we did not find a study that examined the relationship between perceived social support, self-compassion, and self-handicapping. However, according to our observations, having high self-compassion enables nursing students to encourage themselves in a kind, loving, and patient manner when in painful and distressful emotional states. Since self-compassion affects some psychological variables, it is a concept that should be considered within the scope of nursing education.

We identified a significant difference between the mother’s education status of the participants and the CD-RISC scores. Some studies have stated that the education level of parents is an important factor in raising children and adolescents as individuals with strong resilience ^32^. In contrast, mothers with low education raise their children with strict discipline, negatively affecting their resilience ^33^.

The relationship between the scales’ scores of the participating nursing students is supported by studies that reported moderate or high resilience and self-compassion ^15^^,^^27^^,^^34^^,^^35^. Similar to reports in the literature, we found that the self-handicapping tendencies of students were also high ^6^^,^^16^. A significant negative relationship was found between the Self-Handicapping Scale scores and the CD-RISC subdimensions of tenacity and personal competence, tolerance of negative affect, and tendency towards spiritual items. These results suggest that as the level of resilience increases, the tendency of self-handicapping decreases among the students. During undergraduate education, nursing students encounter many stressors related to the academic and clinical environments ^4^^,^^19^^,^^21^. Some individuals can use effective coping strategies to protect themselves and gain psychological strength by turning difficult situations into opportunities ^4^^,^^6^. However, some cannot cope with such events effectively and may self-handicap to legitimize their failure when facing stressful events ^6^.

A positive and significant correlation was found between the mean scores of the participating students on the CD-RISC and Self-Compassion Scale and its sub-dimensions. According to the results, as the resilience of the students increases, their self-compassion also rises. This positive and significant relationship has been reported in other studies ^36^^-^^38^. In conclusion, self-compassion is a protective factor that enhances resilience. In addition, when we examined the relationship between the scores of the Self-Handicapping and the Self-Compassion Scales, we found a significant negative correlation between them and some of their sub-dimensions. Considering this result, as students’ self-compassion increases, their tendency to self-handicapping decreases. Similarly, the literature review revealed that self-handicapping negatively correlates with self-compassion ^25^.

We determined that psychological resilience and self-compassion explained 22 and 36.9% of the variance in self-handicapping among nursing students, respectively. The effect of both scales explained 30.2% of the variance. We observed that as psychological resilience and self-compassion increase among students, their self-handicapping behaviors decrease. A review of the literature indicated that high self-handicapping is related to a tendency to shame, low self-esteem, and low resilience ^25^^,^^26^^,^^39^^-^^41^. In a similar study, Yildirim and Demir found that self-esteem explained 30% of the self-handicapping variance ^25^. The inverse relationship between these two variables can be best explained by considering the motivation of selfhandicap or with low self-esteem individuals to protect themselves.

Our study results suggest that most predictor variables of the scales significantly contribute to explaining the self-handicap variable. Nurses are the most affected occupational group by work stress and burnout among health professionals. This status is due to stressors, such as working conditions, workload, multiple roles, collaborating with different disciplines, caring for many patients in a limited time, and making critical decisions ^4^^,^^42^. Inexperienced nurses -new to the profession- are more affected than experienced nurses ^39^^,^^43^. During their education, nursing students can cope with age-specific individual problems and stressors they face in the academic and clinical environment. Thus, nursing students should be strongly supported in all aspects to prepare them for the profession.

Since the study was conducted in a single province, the results cannot be generalized to all nursing students. The small number of students included is another limitation of this study.

Psychological resilience and self-compassion are qualities that nurses should have when helping people with health problems. By increasing the psychological resilience and self-compassion levels of nursing students -who will work as health care professionals-, they can ensure better quality care by strengthening effective coping skills. Based on the results of this research, we consider important to include topics in the curriculum involving effective coping methods to enhance psychological resilience and self-compassion.

People with high self-compassion accept positive and negative feedback because they feel less threatened by the assessments of themselves ^8^. However, self-handicapped people perceive negative feedback as a threat and avoid facing their failures to protect themselves; they even may attribute those situations to others. In this context, considering the psychological needs of nursing students can help them increase their self-compassion, preventing them from externalizing their failures. Additionally, we suggest creating various programs and intervention studies that could contribute to more compassionate and flexible behaviors toward healthcare providers and others.

## References

[1] 1 Connor KM, Davidson JR. Development of a new resilience scale: The Connor Davidson Resilience Scale (CD-RISC). Depress Anxiety. 2003;18:76-82. 10.1002/da.10113 12964174

[2] 2 Amsrud KE, Lyberg A, Severinsson E. Development of resilience in nursing students: A systematic qualitative review and thematic synthesis. Nurse Educ Pract. 2019;41:102621. 10.1016/j.nepr.2019.102621 31726329

[3] 3 Güngörmüş K, Okanli A, Kocabeyoglu T. Factors influencing resilience in nursing students. J Psychiatr Nurs. 2015;6:9-14. 10.5505/phd.2015.80299

[4] 4 Nam JH, Park HS. The impacts of perceived stress and self-compassion on quality of life of nursing students. J Korean Acad Soc Nurs Educ. 2020;26:67-77. 10.5977/jkasne.2020.26.1.67

[5] 5 Berglas S, Jones E.E. Drug choice as a self-handicapping strategy in response to noncontingent success. J Pers Soc Psychol. 1978;36:405-17. 10.1037/0022-3514.36.4.405 650387

[6] 6 Zarshenas L, Jahromi LA, Jahromi MF, Manshadi MD. Self-handicapping among nursing students: An interventional study. BMC Med Educ. 2019;19:1-7. 10.1186/s12909-018-1441-6 PMC6442433 30929643

[7] 7 Akin Ü, Akin A, Abaci R. Self-Compassion Scale: The study of validity and reliability. Hacet Üniv Eglt Fak Derg. 2007;33:1-10.

[8] 8 Hagerman LA, Manankil-Rankin L, Schwind JK. Self-compassion in undergraduate nursing: An integrative review. Int J Nurs Educ Scholarsh. 2020;17:20200021. 10.1515/ijnes-2020-0021 33151177

[9] 9 Steen M, Othman SME, Briley A, Vernon R, Hutchinson S, Dyer S. Self-compassion education for health professionals (nurses and midwives): Protocol for a sequential explanatory mixed methods study. JMIR Res Protoc. 2022;11:34372. 10.2196/34372 PMC8796041 34848389

[10] 10 Karairmak Ö. Establishing the psychometric qualities of the Connor-Davidson Resilience Scale (CD-RISC) using exploratory and confirmatory factor analysis in a trauma survivor sample. Psychiatry Res. 2010;79:350-6. 10.1016/j.psychres.2009.09.012 20493533

[11] 11 Jones EE, Rhodewalt F. The Self-Handicapping Scale. Princeton, NJ: Princeton University; 1982.

[12] 12 Neff KD. The development and validation of a scale to measure self-compassion. J Pers Assess. 2003;2:223-50. 10.1080/15298860309027

[13] 13 Köklü N, Büyüköztürk S, Qokluk-Bökeoglu Ö. Statistics for social sciences. Ankara: Pegem Publishing; 2015.

[14] 14 Bulduk S, Ardig E. Investigation of self-compassion among nursing students. JAREN. 2015;1:60-5. 10.5222/jaren.2015.060

[15] 15 Qahin G, Buzlu S. The mediating role of perceived stress on relationship of resilience with self-efficacy social support and the effective coping skills in nursing students. J Anatolia Nurs Health Sci. 2017;20:122-35.

[16] 16 Topal S, Qaka SY, Qinar N. Determination ofthe relationship between self-handicapping and burnout of nursing students. Cukurova Univ Educ J. 2018;47:337-56. 10.14812/cuefd.380804

[17] 17 Hanna LA, Clerkin S, Hall M, Craig R, Hanna, A. The resilience of final-year pharmacy students and aspects of the course they found to be resilience-building. Pharmacy. 2022;10:84. 10.3390/pharmacy10040084 PMC9326534 35893722

[18] 18 Taylor H, Reyes H. Self-efficacy and resilience in baccalaureate nursing students. Int J Nurs Educ Scholarsh. 2012;9:1-13. 10.1515/1548-923x.2218 22499714

[19] 19 Ozsaban A, Turan N, Hatice K. Resilience in nursing students: The effect of academic stress and social support. Clin Exp Health Sci. 2019;9:69-76. 10.33808/marusbed.546903

[20] 20 Demiray A, Kiziltepe SK, Agil A, ilaslan N. Determining the stress sources of nursing students. JERN. 2021;18:10-7. 10.5152/jem.2021.68878

[21] 21 Chow KM, Tang WKF, Chan WHC, Sit WHJ, Choi KC, Chan S. Resilience and well-being of university nursing students in Hong Kong: A cross-sectional study. BMC Med Educ. 2018;8:1-8. 10.1186/s12909-018-1119-0 PMC5767064 29329529

[22] 22 Göger S, Qevirme A. The effect of nursing students’ self-efficacy levels on education stress. JERN.2019; 16:306-12. 10.5222/HEAD.2019.306

[23] 23 Nazik E, Arslan S. The investigation of the relations between empathic skills and self-compassion of the nursing students. J Anatolia Nurs Health Sci. 2011;14:69-75.

[24] 24 Sodeify R, Tabrizi FM. Nursing students’ perceptions of effective factors on mental health: A qualitative content analysis. Int J Community Based Nurs Midwifery. 2020;8:34. 10.30476/IJCBNM.2019.81316.0 PMC6969953 32039278

[25] 25 Yildirim Barutgu F, Demir A. Self-handicapping among university students: The role of procrastination, test anxiety, self-esteem, and self-compassion. Psychol Rep. 2020;123:825- 43. 10.1177/0033294118825099 30665332

[26] 26 Gömez-Urquiza JL, Velando-Soriano A, Membrive-Jimenez MJ, Ramirez-Baena L, Aguayo- Estremera R, Ortega-Campos E, *et al*. Prevalence and levels of burnout in nursing students: A systematic review with meta-analysis. Nurse Educ Pract. 2023;72:103753. 10.1016/j.nepr.2023.103753 37651959

[27] 27 Özpulat F, Günaydin N. Nursing students’ self- compassion and their opinions on nursing profession. Int Ref J Nurs Res. 2018;12:40-60.

[28] 28 Pinar ŞE, Bilgig D, Demirel G, Akyüz MB, Karatepe C, Sevim D. Relationship between burnout and life satisfaction of university students in the health field. TAF Prev Med Bull. 2015;14:284-92. 10.5455/pmb.1-1417432935

[29] 29 Ditzen B, Heinrichs M. Psychobiology of social support: the social dimension of stress buffering. Restor Neurol Neurosci. 2014;32:149-62. 10.3233/rnn-139008 23603443

[30] 30 Hostinar CE, Gunnar MR. Social support can buffer against stress and shape brain activity. AJOB Neurosci. 2015;6:34-42. 10.1080/21507740.2015.1047054 PMC4607089 26478822

[31] 31 Terzi Ş. The relationship between psychological hardiness and perceived social support of university students. Turk Psychol Couns Guid J. 2008;3:1-11.

[32] 32 Yavuz K. Psychological resilience in children and adolescents: The power of self-recovery. Curr Approaches Psychiatry. 2023;15:112-31. 10.18863/pgy.1054060

[33] 33 Kurt ŞH, Aslan D. Investigation of self-efficacy, psychological resilience and parental attitudes of preschool children. KADEM Womens Stud. 2020;6:211-40. 10.21798/kadem.2021266729

[34] 34 Al Omari O, Al Yahyaei A, Wynaden D, Damra J, Aljezawi M, Al Qaderi M, *et al*. Correlates of resilience among university students in Oman: A cross-sectional study. BMC Psychol. 2023;11:2. 10.1186/s40359-022-01035-9 PMC9817347 36604764

[35] 35 Larcombe W, Ryan T, Baik C. Are international students relatively resilient? Comparing international and domestic students’ levels of self-compassion, mental health and wellbeing. HERD. 2024;43:362-76. 10.1080/07294360.2023.2234315

[36] 36 Kotera Y, Cockerill V, Chircop J, Kaluzeviciute G, Dyson S. Predicting self-compassion in UK nursing students: Relationships with resilience, engagement, motivation, and mental wellbeing. Nurse Educ Pract. 2021;51:102989. 10.1016/j.nepr.2021.102989 33607377

[37] 37 Ghoncheh S, Golpour R. The relationship between resilience and social support with social health in students: The moderating role of self-compassion. Soc Psychol Res. 2022;12:45- 64. 10.22034/spr.2022.318792.1703

[38] 38 Dilmag Pinar Ş, Ceylan B. Determination of psychological resilience and self-compassion levels of nursing students. JGEHES. 2024;6:1-18. 10.51123/jgehes.2024.108

[39] 39 Yildirim FB, Demir A. The role of self-esteem, self-compassion, and academic self-efficacy in predicting self-handicapping. Ege J Educ. 2017;2:676-701.

[40] 40 Török L, Szabó ZP, Boda-Ujlaky J. Self-esteem, self-conscious emotions, resilience, trait anxiety, and their relation to self-handicapping tendencies. Rev Psychol. 2014;21:123-30.

[41] 41 Aydin A, Kahraman N. Self-handicapping in nursing students: Effects on psychological needs and self-compassion. Cukurova Med J. 2020;45:1625-33. 10.17826/cumj.748170

[42] 42 Duarte J, Pinto-Gouveia J. The role of psychological factors in oncology nurses’ burnout and compassion fatigue symptoms. Eur J Oncol Nurs. 2017;28:114-21. 10.1016/j.ejon.2017.04.002 28478848

[43] 43 Molina-Praena J, Ramirez-Baena L, Gómez-Urquiza JL, Cañadas GR, De la Fuente EI, Cañadas-De la Fuente GA. Levels of burnout and risk factors in medical area nurses: A meta-analytic study. Int J Environ Res Public Health. 2018;15:2800. 10.3390/ijerph15122800 PMC6313576 30544672

